# Surgical approach to management of oroantral communications. Case report

**DOI:** 10.1093/jscr/rjae700

**Published:** 2024-11-16

**Authors:** Edgar Salas, Luis Gabriel Ladino

**Affiliations:** Private Practice in Oral Surgery, Calle 13 # 1E - 81 los Caobos, Cúcuta, Colombia; Institución Universitaria Colegios de Colombia UNICOC, Km 20, Autonorte I-55, Chía, Cundinamarca, Colombia; Universidad Católica del Uruguay, Av. 8 de Octubre 2738, 11600 Montevideo, Uruguay

**Keywords:** oroantral communication, oral surgery, maxillary sinusitis, oroantral fistula, maxillary sinus

## Abstract

One of the most frequent post-extraction complications of posterior maxillary teeth is oroantral communication, which consists of direct communication between the maxillary sinus and the oral cavity. The recommendation is to make an immediate closure within the first 48 hours to avoid possible complications that may include an oroantral fistula or chronic sinus disease. Currently, different techniques have been described for closing communication, including various types of materials. Some of the techniques are exclusively oriented toward closure, while others seek to achieve bone regeneration by using bone substitutes for the subsequent placement of dental implants. The aim of this study is to describe a simple surgical technique for managing oroantral communication using a collagen sponge with hydroxyapatite and ribose and covering it with overlapping double layers of ribose cross-linked collagen membrane without fixation.

## Introduction

Oroantral communication (OAC) is the space created between the maxillary sinus and the oral cavity [1]. The most common cause is the extraction of maxillary posterior teeth. Maxillary second molar extractions cause 45% of communications, third molars cause 30%, first molars cause 27.2%, and first premolars cause 5.3%. Additionally, 2.2% of first molar apices and 2% of second molar apices perforate the maxillary sinus floor [2]. Another cause includes tumor surgery, implant surgery, trauma, or orthognathic surgery that involves the maxilla [3]. Although not all communications require surgical closure because some can heal spontaneously; untreated communications could induce a fistula by bacteria colonization or chronic sinusitis. In order to avoid these complications, it is recommended that communication be closed within 48 hours. There are different options to get communication closure; some of them are conservative techniques, such as suturing the gingiva of buccal flaps, and others are invasive, such as pedicled buccal fat pads, modified submucosal connective tissue flaps, distant flaps, autogenous bone grafts, allogenous synthetic materials and metals, and other techniques [4]. However, there is no best treatment or consensus regarding oroantral communication. Each technique has its advantages and disadvantages. The choice of a method must be made on a case-by-case basis, considering all factors regarding the act and the patient [5, 6]. The aim of this study is to describe a simple surgical technique for managing oroantral communication using a collagen sponge and covering it with overlapping double layers of ribose-cross-linked collagen membrane without fixation. A clinical case is described in detail with 1 year of follow-up, including a CT scan and clinical control. In addition, a bone sample was taken for histological evaluation 6 months after surgery.

## Case report

**Figure 1:**
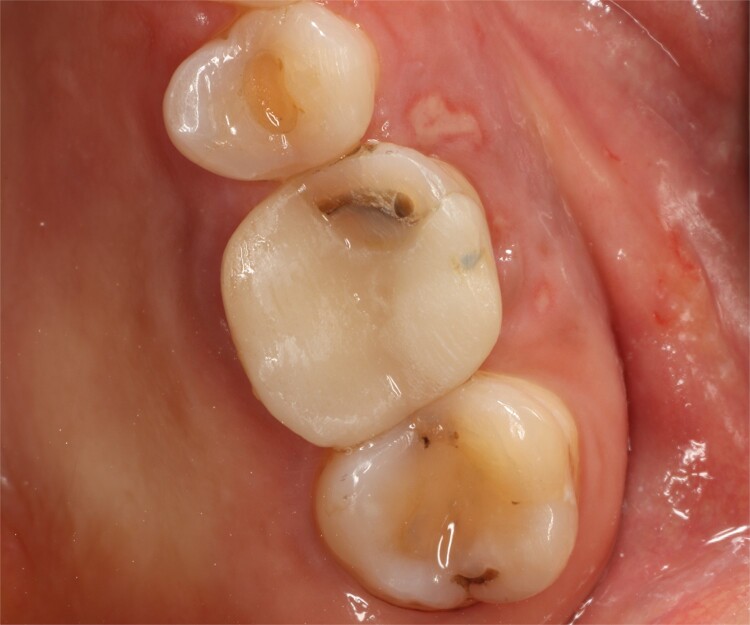
Preoperative intraoral view.

**Figure 2:**
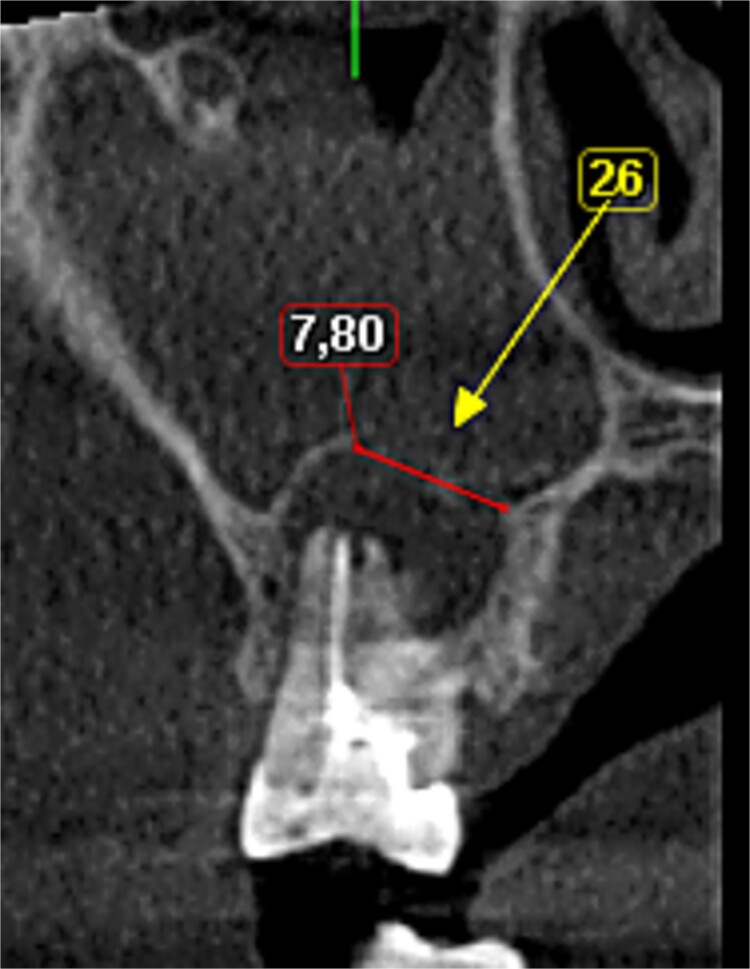
Preoperative CBCT view.

A 56-year-old female patient without any systemic diseases was referred by otolaryngology for evaluation by oral surgery due to presenting pain on the left side of the face for two weeks. The patient’s first molar presented a wide restoration (Fig. 1). This molar had undergone endodontic treatment some years ago. The CT scan showed a hyperdense left maxillary sinus almost entirely with loss of continuity of the maxillary sinus floor at the level of the left upper first molar involving the furcation zone, it is evident an oroantral communication of 7 mm (Fig. 2). The patient referred to spontaneous pain during chewing. Based on the CT scan and the clinical findings, pharmacological management was indicated with oral antihistamine 10 mg every 24 hours for 10 days, Moxifloxacin 400 mg every 24 hours, and scheduled extraction with oroantral communication closure with collagen sponge on the third day after starting the antibiotic, which will be used for a total of 10 days. The patient agreed to sign an informed consent file to make the technique to extraction and close the oroantral communication.

**Figure 3:**
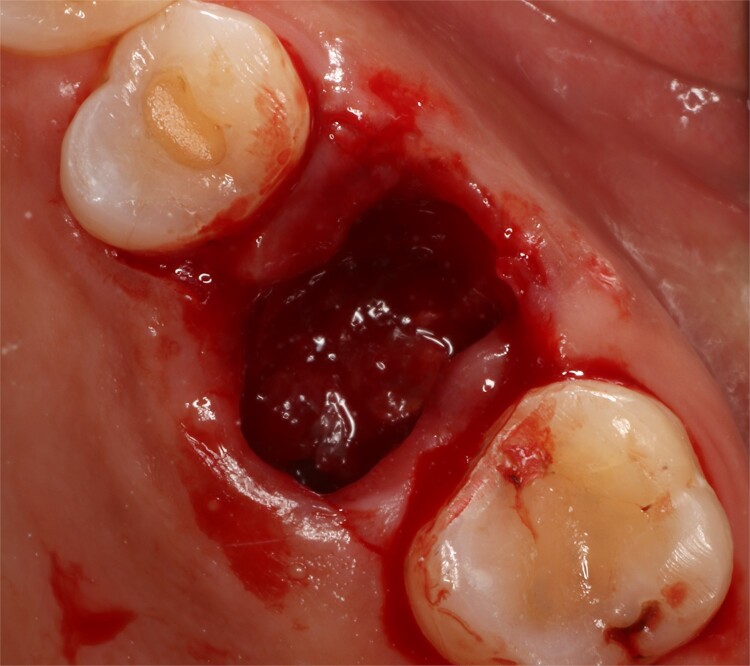
Socket view after extraction.

**Figure 4:**
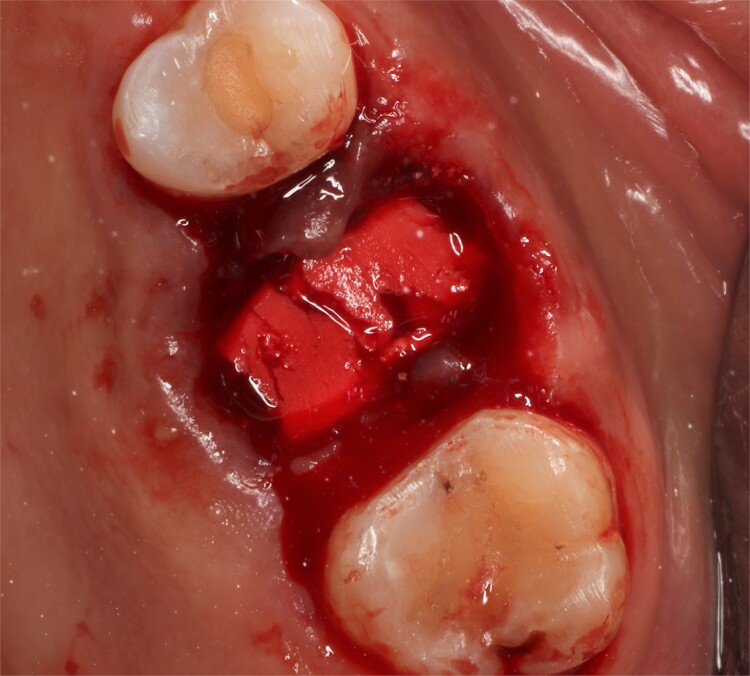
Collagen sponge inside the socket.

**Figure 5:**
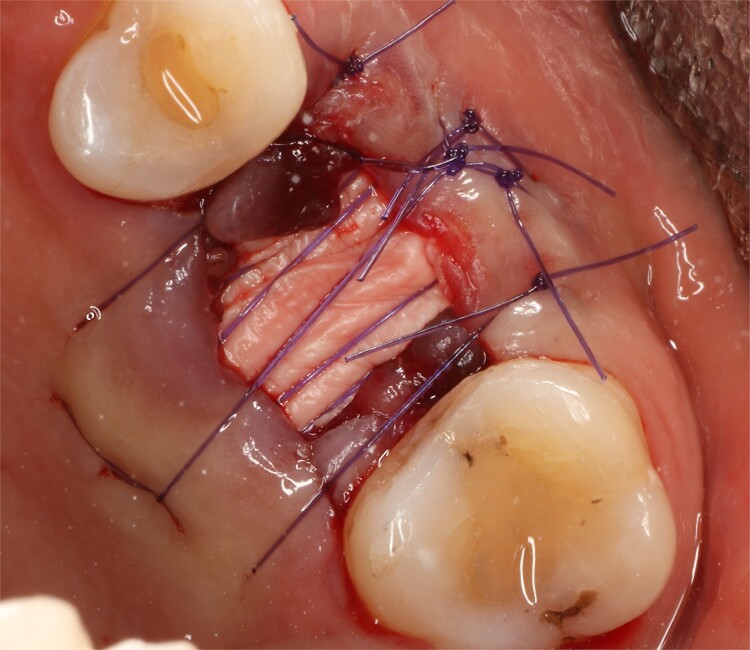
Stabilization of collagen membrane.

Following local anesthesia, an intrasulcural incision was made from the distal surface of the premolar, continuing through the sulcus to the mesial surface of the second molar in the vestibular and palatal surfaces. After the molar was extracted, granulation tissue was observed inside the socket that continued inside the maxillary sinus (Fig. 3); The socket was scaled, washed, and rinsed with physiological saline. The labial and palatal mucoperiosteal flaps are raised with full-thickness tunneling of the tissue, exposing the labial and palatal bone plates to allow a space for the ribose-crosslinked collagen membrane that will be used for socket closure. A 5 mm × 10 mm × 10 mm collagen sponge with hydroxyapatite and ribose was used, which was taken to the alveolus without hydration so that it absorbs as much blood as possible. It is important that the sponge has retention in the alveolus so that it does not displace within the maxillary sinus and is positioned at the same level as the remaining bone crest. A collagen membrane crosslinked with ribose was placed from buccal to palatal under the tunneled tissues (Fig. 4). A 5–0 absorbable monofilament suture was used, another layer of the collagen membrane was used in the occlusal area that was exposed, and two horizontal mattresses were sutured to give stability to the biomaterials (Fig. 5).

**Figure 6:**
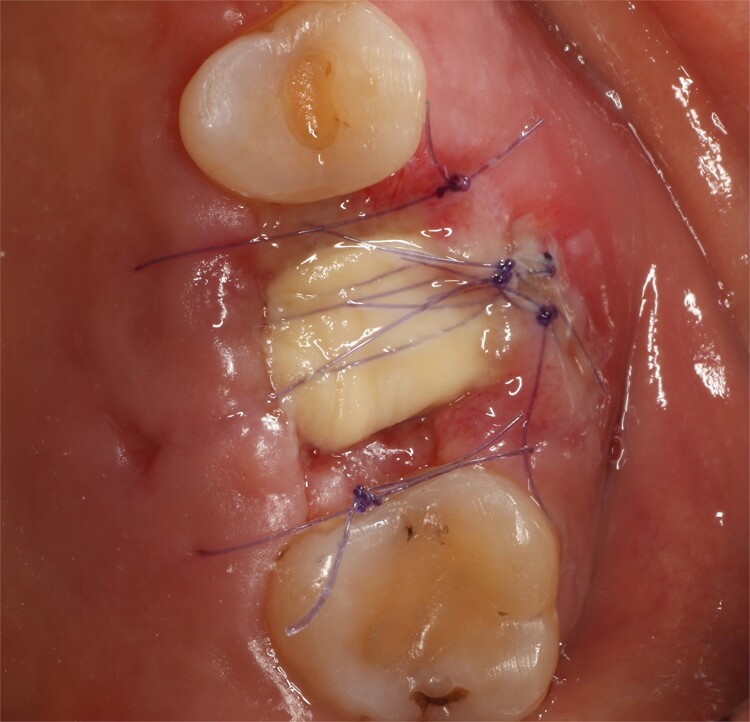
Follow-up at 8 days after surgery.

**Figure 7:**
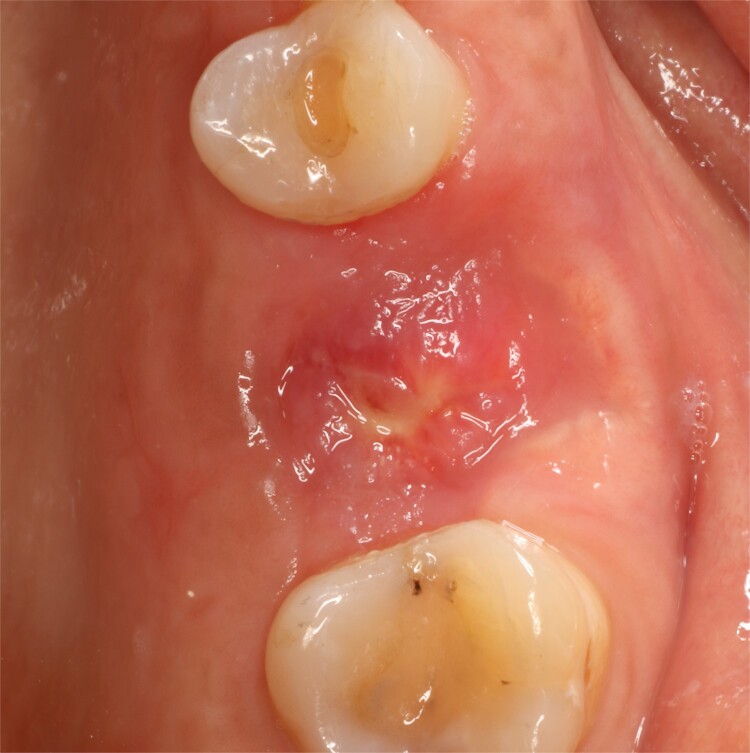
Follow-up at 4 weeks after surgery.

**Figure 8:**
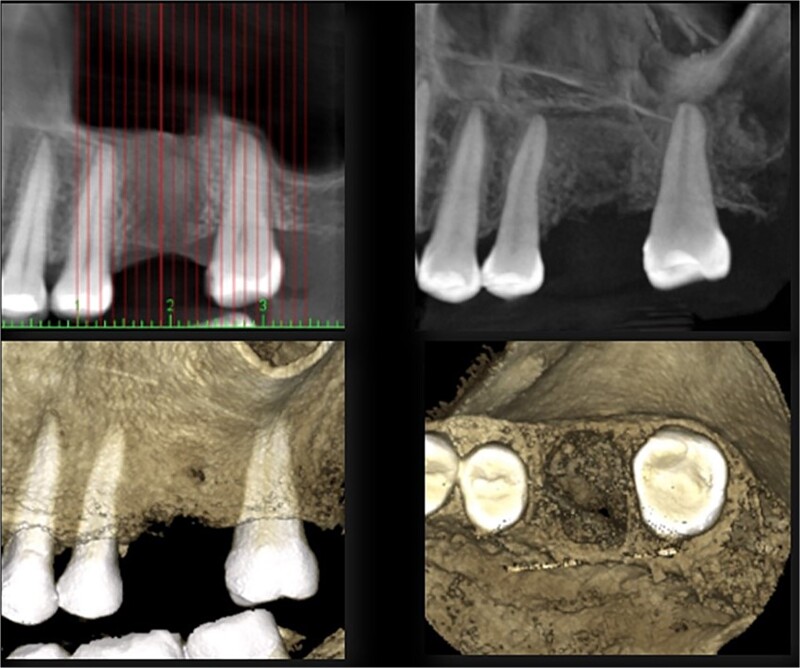
CBCT 3 months after surgery.

**Figure 9:**
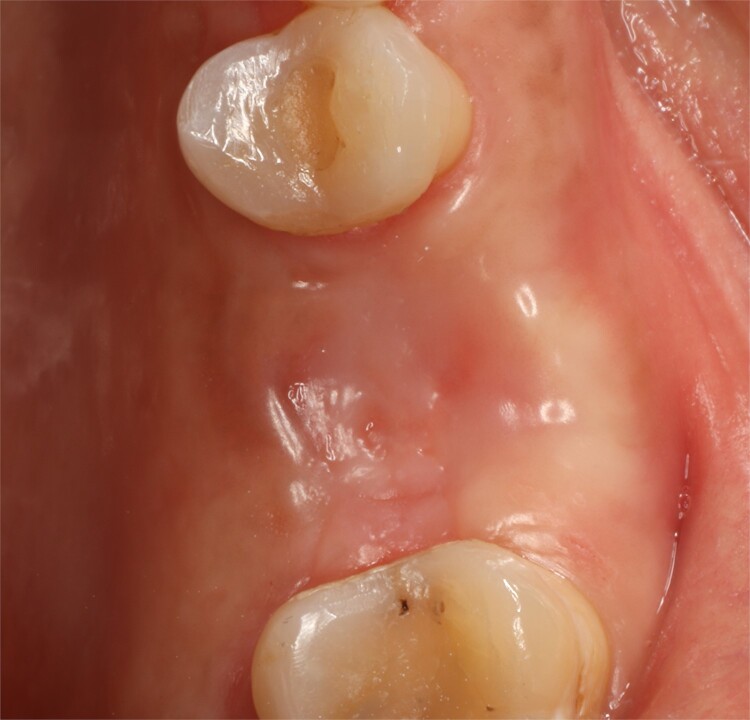
Follow-up at 6 months after surgery.

**Figure 10:**
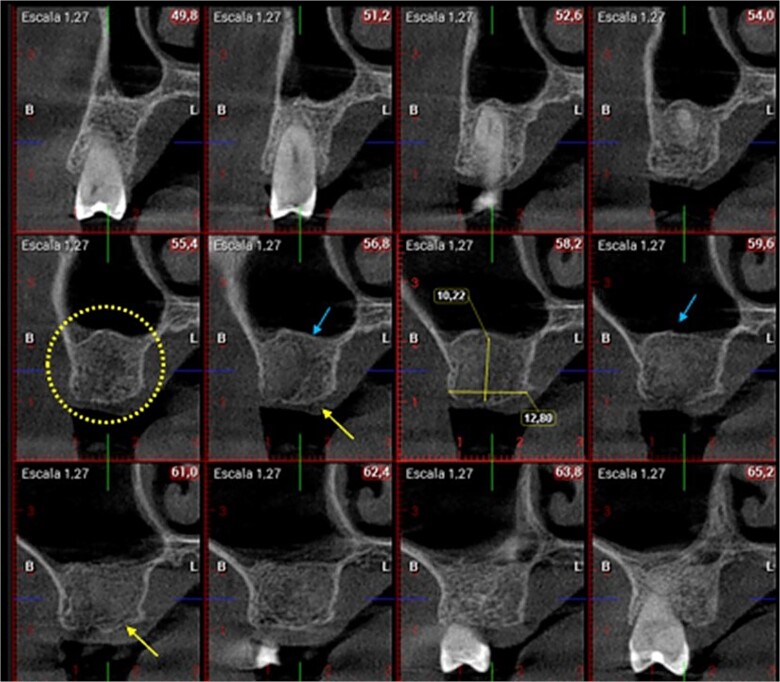
CBCT view 12 months after surgery.

A control appointment was made 8 days later, and the scar tissue with slight erythema on the edges of the alveolus was observed (Fig. 6). The patient was asymptomatic, and the membrane was in position to cover the entire alveolus. After the fourth week, granulation tissue was observed covering almost the entire alveolus, and an area was observed where the membrane found below this granulation tissue could be seen (Fig. 7). About 3 months after surgery, healed soft tissue was observed, and a CT scan was requested, where the maxillary sinus was observed without evidence of pathologies and hypodense alveolar bone compatible with immature bone in the process of calcification (Fig. 8). After 6 months, keratinized tissue and a completely healed alveolus were observed (Fig. 9), and cone beam computed tomography (CBCT) was requested to evaluate the results and see the possibility of placing an implant. In the CT scan, continuous sinus and alveolar cortex were observed with an image compatible with bone in the healing process (Fig. 10).

## Discussion

The ideal treatment for oroantral communication must be fast, secure, simple, and well tolerated by patients, at low cost, resulting in good bone and mucosal healing [7]. The classic closure techniques with flap elevation, can carry the frequent disadvantage of second intention healing and soft tissue topography alteration [8], the reduction of the vestibular depth could compromise the stability of final restoration, in addition, the positioning and the suturing of the flap may require a long time to be learned [9].

Crosslinked collagen membranes stay intact sufficiently long to achieve early periodontal wound healing. For instance, crosslinked porcine collagen membranes were found six months after guided bone regeneration. The use of ribose, a natural and non-toxic crosslinking agent, effectively extends resorption time for up to 16 to 24 weeks while resulting in sufficient permeability to allow progenitor cells’ migration and giving it greater resistance to degradation when exposed for a period of 3 to 6 weeks [6]. Due to its characteristics, this collagen sponge can help alveolar closure and regeneration in contained defects such as this clinical case, but when this membrane is lost, part of the vestibular and palatal bone walls will be lost, and the socket will contract significantly proportional to the bone loss. When this membrane is stable with suture, the formation of keratinized tissue is more significant than if primary closure had been performed, since when confronting the flaps, the possibility of gaining tissue is reached. In addition, it avoids the depth of the vestibule and gingival architecture being altered, which would force a few months later to perform surgical procedures to restore the depth of the vestibule and create keratinized gingiva. The use of these sponges has proven to be an efficient way to preserve and regenerate alveoli without leaving evidence of biomaterial in histological sections performed after 6 to 8 months of healing [6, 10]. A recent systematic review showed that the use of platelet-rich fibrin in the treatment of oroantral communications/fistulas has several advantages because it helps maintain the position of the mucogingival junction without requiring the coronal displacement of mucoperiosteal flaps [11]. The criteria for selecting the technique for closure of OAC include choosing the proper biomaterial, the size of communication, time of diagnosis, and improper treatment of sinus infection preoperatively, all of these to avoid complications [4].

## Conflict of interest statement

None declared.

## Funding

None declared.
